# Comparative efficacy and safety of targeted therapy and immunotherapy for HER2-positive breast cancer: a systematic review and network meta-analyses

**DOI:** 10.3389/fonc.2024.1331055

**Published:** 2024-04-03

**Authors:** Suyu Gu, Yuting Liu, Yufan Huang, Wenzheng Lin, Ke Li

**Affiliations:** ^1^ Institute of Translational Medicine, Medical College, Yangzhou University, Yangzhou, China; ^2^ Affiliated Hospital of Yangzhou University, Yangzhou University, Yangzhou, China; ^3^ Department of Eighth Internal Medicine, Shenyang Traditional Chinese Medicine Hospital, Shenyang, China

**Keywords:** HER2-positive, breast cancer, immunotherapies, efficacy, safety, meta-analysis

## Abstract

**Background:**

In recent years, novel therapies targeting specific molecular pathways and immunotherapies have exhibited promising outcomes for treating human epidermal growth factor receptor 2 (HER2)-positive breast cancer. Our work aimed to assess the effectiveness and safety of these emerging treatment regimens for this disease.

**Material and methods:**

We systematically searched databases including PubMed, Embase, Web of Science, and the Cochrane Central Register of Controlled Trials their inception to August 2023 to identify relevant randomized controlled trials (RCTs). The quality of eligible RCTs was evaluated with the Cochrane risk-of-bias tool, version 2 (RoB2). Investigated outcomes encompassed progression-free survival (PFS), overall survival (OS), disease-free survival (DFS), pathologic complete remission (pCR), and adverse events (AEs). They were expressed as hazard ratio (HR) with 95% conference intervals (CI) or risk ratio (RR) with 95% CI.

**Results:**

Our analysis identified a total of 28 RCTs suitable for inclusion in the NMA. Regarding the PFS, all these treatment regimens exhibited comparable effectiveness. In terms of OS, Capecitabine+Trastuzumab, Lapatinib+Trastuzumab and Pyrotinib+Capecitabine exhibited better effect compared to other treatments. Regarding pCR and AEs, all these treatment regimens exhibited comparable effectiveness, especially Lapatinib+Trastuzumab and Pyrotinib+Capecitabine.

**Conclusion:**

Our study highlights the prominent role of targeted therapies and immunotherapies in treating HER2-positive breast cancer. The efficacy of trastuzumab-containing regimens was superior to other treatment options, while maintaining a comparable safety profile. Based on these findings, trastuzumab-containing regimens emerge as a preferable and recommended choice in clinical practice for managing HER2-positive breast cancer.

**Systematic Review Registration:**

PROSPERO, identifier CRD42023414348.

## 1 Introduction

Breast cancer constitutes a significant threat to women’s health, ranking prominently among the leading causes of mortality in postmenopausal women. It accounts for a significant portion, up to 23%, of all cancer-related deaths, underscoring its profound global public health significance (1). Human epidermal growth factor receptor 2 (HER2), a member of the erythroblastic oncogene B (ErbB) family of receptor tyrosine kinases, underpins the development of breast cancer (BC) (2). Remarkably, around 25% of BC patients exhibit positive HER2 expression, signifying the clinical relevance of this molecular marker (3). Research by Slamon DJ, et al. has indicated that HER2 positivity correlated with heightened metastatic potential and a less favorable prognosis, further emphasizing the importance of HER2 as a prognostic factor (4). Consequently, the inhibition of HER2 has emerged as a promising avenue for therapeutic intervention, with the potential to curtail the growth of HER2-positive BC and offer new hope to affected individuals (2).

The preferred first-line therapy for HER2-positive BC currently is the combination of trastuzumab, pertuzumab, and paclitaxel (THP) (5). Recent decades have witnessed mounting evidence to support the significance of the immune system in regulating treatment response and survival outcomes among BC patients (6). The potential of immune checkpoint blockade therapy in managing BC has shown promise, highlighting its ability to leverage the immune system for clinical benefits. Consequently, targeted therapies and immunotherapy are anticipated to assume greater significance in standard treatment approaches, offering novel directions and renewed hope for the future management of breast cancer.

Network meta-analysis (NMA) presents a superior approach compared to traditional methods as it allows for simultaneous comparisons of multiple treatments while considering various potential sources of heterogeneity, potentially identifying the optimal treatment strategy more effectively. Randomized controlled trials (RCTs) have substantially aided in the integration of innovative therapeutic strategies into clinical practice over the past decade. This is especially true for the incorporation of new targeted therapies alongside immunotherapy for HER2-positive BC. As a result, leveraging RCT data serves as a robust foundation for supporting our current study.

Targeted therapies and immunotherapies have emerged as crucial components in the post-surgery, radiotherapy, and anti-tumor chemotherapy treatment landscape for malignancies (7). Given the ever-evolving interactions within immune signaling pathways and the immune microenvironment, there exists a wide array of immune-targeted drugs available. However, specific comparisons regarding their corresponding anti-tumor effects are often lacking. As a response to this gap, we performed this NMA to provide a comparison of the efficacy and safety of current immunotherapies and targeted therapies for HER2-positive BC, with the aim of providing valuable evidence to inform treatment decisions.

## 2 Materials and methods

This NMA was reported following the Preferred Reporting Items for Systematic Review and Meta-Analysis (PRISMA) extension statement for systematic reviews incorporating NMAs (8). The prespecified protocol was registered on the International Prospective Register of Systematic Reviews (PROSPERO) with a registration number (CRD42023414348).

### 2.1 Literature search

Th literature search was performed in databases including PubMed, Embase, Web of Science, and the Cochrane Central Register of Controlled Trials. The search targeted RCTs related to targeted therapies and immunotherapies for HER2-positive BC, spanning from the inception of these databases to August 9, 2023. We imposed no restrictions regarding publication date or language.

### 2.2 Selection criteria

Studies should meet the following criteria for inclusion in this NMA: (1) study design: RCT; (2) Population: patients were diagnosed with HER2-positive BC; (3) Intervention: targeted therapies or immunotherapies (4) Outcomes: progression free survival (PFS), overall survival (OS), disease free survival (DFS), pathologic complete response (pCR), and adverse events.

### 2.3 Data extraction and quality assessment

Using a pilot-tested data extraction form, three investigators independently extracted data from included RCTs. The extracted information encompassed various aspects, such as the first author, publication year, sample size, treatment regimens, and outcomes (PFS, OS, DFS, and pCR), along with the number of adverse events. Any disparities were resolved through deliberation with the other three reviewers.

The Cochrane risk-of-bias tool was employed for assessing the quality of eligible RCTs (9), evaluating domains like randomization sequence generation; allocation concealment; blinding of participants and personnel, incomplete outcome data, selective outcome reporting, and other sources of bias. Each domain was assessed to rate each RCT as having a low, high, or unclear risk of bias.

### 2.4 Data analysis

We conducted the NMA considering both direct and indirect treatment comparisons to evaluate the effectiveness of the targeted treatment regimens. To gauge the extent of heterogeneity across included RCTs, we employed the *I* ^2^ statistic, with a value exceeding 50% indicating significant heterogeneity (10). The presence or absence of significant heterogeneity led to the application of two distinct models: a random-effects model (11) was selected in cases of substantial heterogeneity, while a fixed-effects model (12) was employed when heterogeneity was not pronounced. Our pooled estimates for PFS and DFS are presented alongside the corresponding hazard ratios (HRs) and corresponding 95% confidence intervals (CI). Dichotomous data, such as pCR and adverse events (AEs), were represented by risk ratios (RRs) and corresponding 95% CIs. Statistically significant findings were identified when both the upper and lower limits of the 95% CI were either greater than or less than one, equivalent to p-values < 0.05, the conventional threshold for statistical significance. We derived P scores as the frequentist equivalent of the Surface under the Cumulative Ranking Curve (SUCRA) (13). Data analyses was performed using the R package “netmeta” (version 1.2.0, R Foundation) (14), enabling us to conduct this extensive analysis of treatment effectiveness and rankings.

## 3 Results

### 3.1 Study identification and characteristics

**Figure 1:**
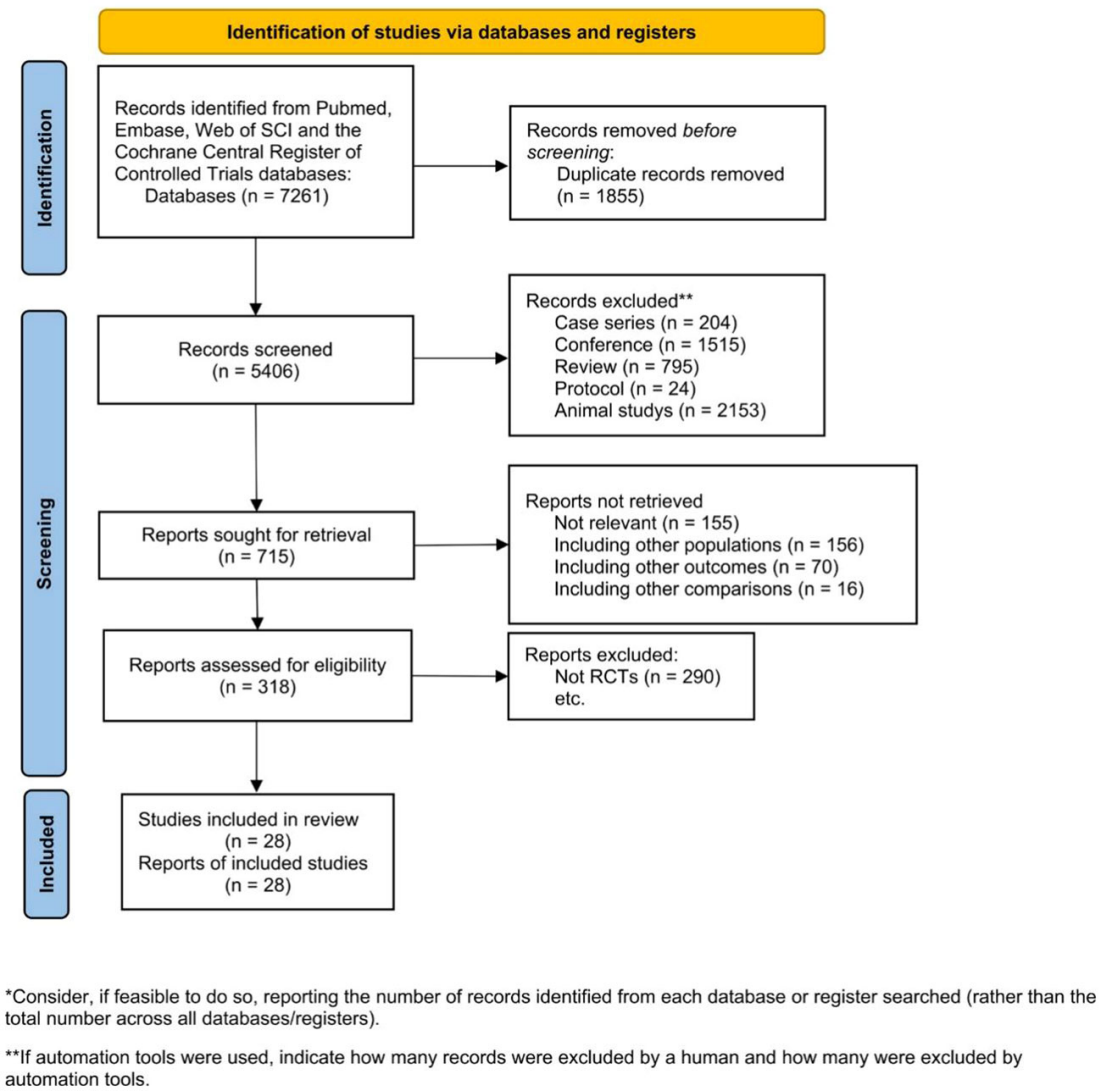
Eligibility of studies for inclusion in meta-analysis.

The initial search identified 7,261 studies, from which 1,855 duplicate records were excluded. Subsequently, the titles and abstracts of 5,406 papers were scrutinized, and 4,691 were excluded for various reasons, including case series (n=204), conference abstracts (n=1,515), reviews (n=795), protocols (n=24), and animal studies (n=2,153). Following this, 715 publications underwent a detailed eligibility assessment through full-text reading. However, 397 of them were excluded for various reasons, leaving 318 articles for further consideration. Among these, 290 were excluded for not meeting inclusion criteria. Finally, 28 RCTs were included in the NMA (**Figure 1**).

Study characteristics are outlined in **Supplementary Table S1**. These RCTs spanned multiple regions, with 14 studies conducted in North America, 6 in China, and several encompassing a global scope. A comprehensive analysis involved 27,464 patients diagnosed with HER2-positive BC, facilitating assessments of OS and pCR. Targeted treatments and immunotherapy regimens were comprehensively compared across all included RCTs, incorporating lapatinib, afatinib, trastuzumab, pertuzumab, neratinib, atezolizumab, lapatinib, abemaciclib, T-DM1and margetuximab. These RCTs were published between 2013 and 2023, providing data on OS and pCR. Patient demographics revealed an age range of 19 to 88 years across all studies, while a median duration of follow-up ranging from 3 to 64 months.

### 3.2 Risk of bias

**Figure 2:**
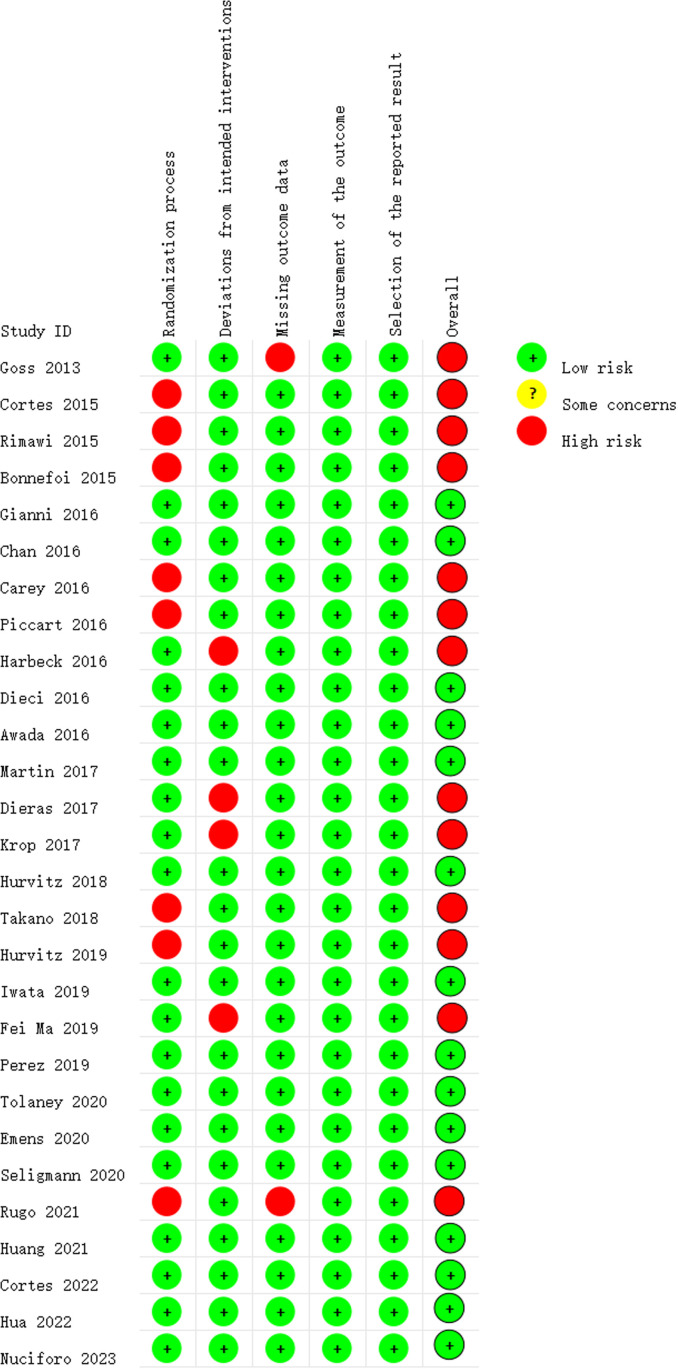
Risk of bias summary.

**Figure 2** depicts the ROB assessment results for RCTs. Among the reviewed studies, 15 were rated as having a low ROB, while 13 were determined to have a high ROB, primarily due to at least one of the five ROB domains being assessed as a ‘high’. Regarding the randomization process, 20 studies reported information on the randomization sequence or concealment, resulting in a low ROB rating. Four studies were scored as having a high ROB in the ‘Deviations from Intended Interventions’ domain. Among these, two studies reported that both participants and implementers were aware of the assigned condition, disclosed deviations from the intended intervention, and conducted appropriate analyses, such as intention-to-treat, to estimate the effect of assignment to conditions. The assessment of incomplete outcome illustrated that all but one study was rated as having a low ROB. The exceptional study failed to provide a reason for the missing data, so it is difficult to determine whether the missing data correlate with the true value in all cases, or whether they might correlate with the true value of the results. All studies were rated as having a low ROB for the assessment of selective reporting.

### 3.3 Networks meta-analysis for outcomes

#### 3.3.1 Primary endpoints: overall survival

**Figure 3:**
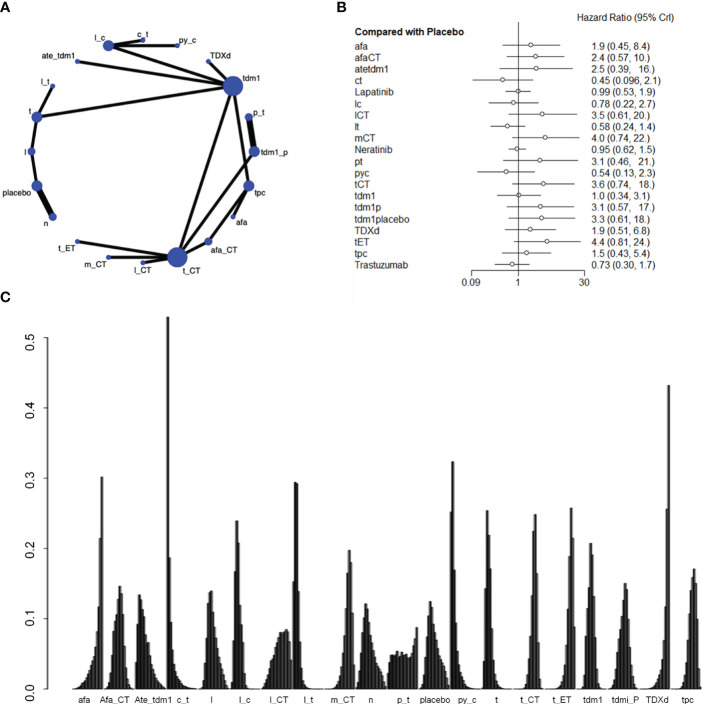
Plots of overall survival (OS). **(A)** Network plot. **(B)** Forest plot. **(C) **Ranking plot.

Nineteen studies involving 21,243 patients and 21 interventional arms analyzed OS. The top three of SUCRA ranking for overall survival were Capecitabine+Trastuzumab (0.924), Lapatinib + Trastuzumab (0.901) and Pyrotinib + Capecitabine (0.900). The overall survival of Capecitabine+Trastuzumab was superior to the rest therapies. In terms of improvement for overall survival(OS), Capecitabine+Trastuzumab was significantly superior to Lapatinib+CT(HR=2.76 (95CI: 1.02 - 7.4)), Margetuximab+CT(HR=3.21 (95CI: 1.33 - 7.84)), Trastuzumab+ET (HR=3.49 (95CI: 1.45 - 8.46)), tdm1(HR=2.29 (95CI: 1.01 - 5.2)), tdm1+Pertuzumab(HR=2.46 (95CI: 1.02 - 6.01)), T-DXd(HR=4.17 (95CI: 1.65 - 10.59)), tpc(HR=2.98 (95CI: 1.28 - 6.95)). Lapatinib + Trastuzumab was significantly superior to Trastuzumab+ET(HR=2.03 (95CI: 1.4 - 2.93)), T-DXd(HR=2.42 (95CI: 1.53 - 3.85)). Pyrotinib + Capecitabine was significantly superior to Trastuzumab+CT(HR=2.41 (95CI: 1.3 - 4.45)), Trastuzumab+ET(HR=2.94 (95CI:1.53 - 5.68)), tdm1+Pertuzumab(HR=2.07 (95CI: 1.07 - 4.02)), T-DXd(HR=3.51 (95CI: 1.72 - 7.18)), tpc(HR=2.51 (95CI: 1.36 - 4.61)). Afatinib (SUCRA=0.143) and Trastuzumab deruxtecan(SUCRA=0.075) indicate poor performance in the results of overall survival. Based on their performance in the league tables, it can also be concluded that those two therapies are significantly less effective than other therapies. When those two drugs are used as necessary, it is important to exercise caution when determining the appropriate dosage (**Figure 3**, **Supplementary Table S2**).

#### 3.3.2 Secondary endpoints: pathologic complete response

**Figure 4:**
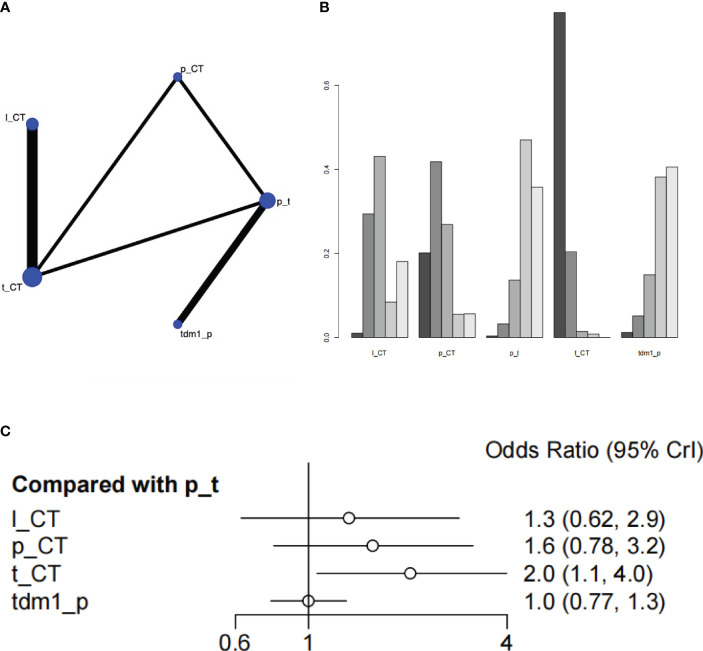
Plots of pathologic complete response (pCR). **(A)** Network plot. **(B)** Ranking plot. **(C)** Forest plot.

Two studies with a total of 1105 patients, containing 5 interventional arms that analyzed the pCR. The top one of SUCRA ranking for pathologic complete response (pCR) was Trastuzumab + chemical therapy. Trastuzumab+CT was significantly superior to Pertuzumab+Trastuzumab(HR=2.03 (95CI: 1.06 - 3.99)). However, Trastuzumab + chemical therapy didn’t have satisfactory performance in the results of overall survival, so it might not be a preferred choice (**Figure 4**, **Supplementary Table S3**).

#### 3.3.3 Adverse events

**Figure 5:**
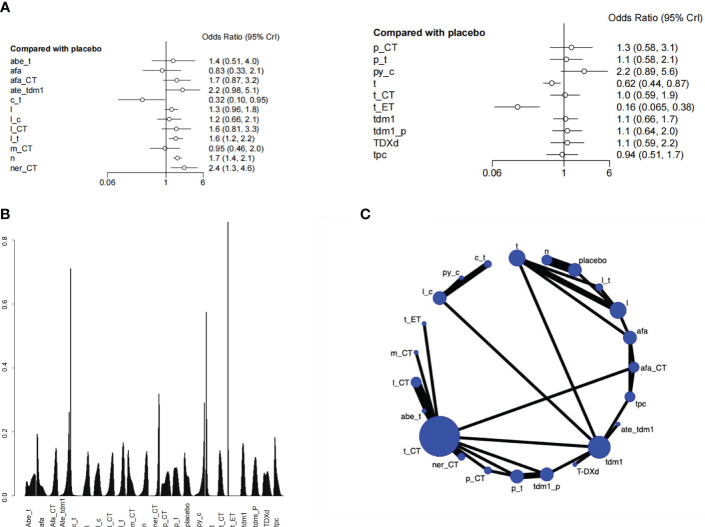
Plots of Adverse events (AEs). **(A)** Forest plot. **(B)** Ranking plot. **(C)** Network plot.

There was a total of 27 RCTs with 25000 patients analyzing the endpoint of AEs. According to the SUCRA ranking, the safety of Pyrotinib + Capecitabine (0.144) and Atezolizumab+Trastuzumab emtansine (0.128) were relatively high. Pyrotinib + Capecitabine was significantly superior to Capecitabine + Trastuzumab (HR=6.91 (95CI: 2.14 - 23.92)). Atezolizumab+Trastuzumab emtansine was significantly superior to Capecitabine + Trastuzumab(HR=6.93 (95CI: 2.13 - 24.41)), Trastuzumab(HR= 3.57 (95CI: 1.71 - 7.68)), Trastuzumab+ET(95CI: 13.74 (5.19 - 38.56)). If patients are particularly concerned about adverse reactions, these drugs especially Pyrotinib+Capecitabine can be given priority. Besides, the safety of Lapatinib + Trastuzumab was also higher than placebo, in view of its excellent overall survival, it can also be a good choice. Although Capecitabine + Trastuzumab ranked high in the SUCRA ranking of overall survival, it didn’t perform well in the ranking of adverse events, which has a higher risk than placebo (**Figure 5**, **Supplementary Table S4**).

## 4 Discussion

### 4.1 Major findings

This NMA has comprehensively evaluated the effectiveness and safety of targeted therapies and immunotherapies available for the treatment of HER2-positive BC. The analysis result indicated that, concurrent use of targeted therapies and immunotherapies has demonstrated noteworthy enhancements in OS for individuals diagnosed with HER2-positive BC. For OS, Capecitabine + Trastuzumab, Lapatinib + Trastuzumab and Pyrotinib+Capecitabine exhibited better effect other treatments. For pCR and adverse event, all these treatments had no significant difference with each other.

### 4.2 Comparison with other reviews

In this meta-analysis, our findings underscore the significant superiority of trastuzumab plus capecitabine in OS when compared to various alternative treatments, including lapatinib + chemotherapy, margetuximab + chemotherapy, pertuzumab + trastuzumab, trastuzumab + chemotherapy, TDM1 + placebo, trastuzumab deruxtecan, and trastuzumab + endocrine therapy. These results underscore the superior survival benefits of trastuzumab for HER2-positive BC, aligning with prior studies (15–17), including the analysis by O’Sullivan CC et al. of five RCTs comparing the effectiveness of trastuzumab among individuals with small (≤ 2 cm) HER2-positive BC (15). Their findings, focusing on hormone receptor (HR)-positive patients with a median follow-up of 8 years, demonstrated considerable benefits in OS and DFS for trastuzumab-treated patients. The study highlighted that cumulative incidence rates favored trastuzumab over no trastuzumab, emphasizing its efficacy for DFS (17.3% versus 24.3%, P < 0.001) and OS (7.8% versus 11.6%, P = 0.005). Similar advantages were observed in patients with HR-negative disease (15). Furthermore, a meta-analysis published in 2023 highlighted the advantageous effects of combining trastuzumab with Tyrosine Kinase Inhibitors (TKIs) in HER2-positive BC (16). Systematically searching databases up to September 2022 and including sixteen RCTs, the authors reported that trastuzumab plus TKI was associated with significantly elevated OS (HR=0.77, 95%CI: 0.67-0.88, P<0.001), PFS (HR=0.52, 95%CI: 0.41-0.66, P<0.001), pCR (OR=1.90, 95%CI: 1.50-2.41, P=0.001), and ORR (OR=2.17, 95%CI: 1.34-3.50, P=0.002) compared to trastuzumab monotherapy (16). These results signify the enhanced efficacy of trastuzumab plus TKI across different stages of HER2-positive BC.

In another NMA focused on investigating the optimal neoadjuvant regimen for HER2-positive BC, researchers discovered that dual anti-HER2 therapy, involving pertuzumab or TKIs, combined with chemotherapy, exhibited significant superiority over trastuzumab and chemotherapy regarding pCR, OS, and Event-Free Survival (EFS) (18). This comprehensive study encompassed 46 RCTs with 11,049 patients diagnosed with HER2-positive BC, evaluating 32 different treatment modalities. The analysis of EFS (n=4919) demonstrated that dual HER2 blockade, either with the combination of pertuzumab + trastuzumab + chemotherapy or TKI + trastuzumab + chemotherapy, demonstrated improved EFS than chemotherapy + trastuzumab (HR=0.61, 95% CI: 0.43–0.85) and T-DM1 (HR=0.66, 95% CI: 0.34-1.26). Furthermore, the combination of dual anti-HER2 blockade and chemotherapy exhibited benefits in pCR, EFS, and OS compared to a single anti-HER2 agent combined with chemotherapy. Dual anti-HER2 combined with chemotherapy was superior to T-DM1 regarding EFS and pCR. Anti-HER2 + chemotherapy outperformed dual anti-HER2 alone regarding pCR and EFS, with a noticeable trend towards better OS. They concluded that dual HER2 blockade plus chemotherapy stands out as the preferred option for neoadjuvant therapy in HER2-positive BC. On one hand, our findings provide more evidence for previous studies. Those findings are in support of previous studies and are supported by newer evidence, adding to their credibility and relevance. On the other hand, the findings indicate that certain research methods require more attention, such as Lapatinib + Trastuzumab and Pyrotinib + Capecitabine. Regarding the safety profile of the various treatment regimens, this study found no significant differences among them. Notably, concerning cardiac safety, there was no increase in the incidence of cardiotoxicity or toxic deaths with trastuzumab-based regimens. It is essential to highlight that the ALTTO study reported more discontinuations due to increased toxicity linked to lapatinib plus trastuzumab compared to trastuzumab monotherapy (19). Specifically, compared to trastuzumab, therapy containing lapatinib exhibited a higher rate of grade ≥ 3 diarrhea (1% vs. 15%), predominantly contributing to treatment discontinuation. In contrast, Murthy et al. (20) found no significant differences in withdrawal rates between patients administered by tucatinib or trastuzumab plus capecitabine. This suggests that safety considerations vary across different treatment combinations, emphasizing the importance of carefully assessing safety profiles when determining treatment strategies.

### 4.3 Advantages and limitations

There are some limitations to our work. Firstly, the quality of the included literature requires improvement, as a majority of studies did not report the random allocation method. Additionally, a substantial number of studies were open-label, potentially introducing an increased risk of bias in the research outcomes. Secondly, the heterogeneity in outcome indicators across studies limits the comprehensiveness of drug effectiveness comparisons. Lastly, the relatively small number of included RCTs and the constraints in sample size may potentially exaggerate the efficacy of carious treatments. These limitations underscore the need for caution in interpreting the study findings and emphasize the importance of further research with improved methodological rigor and larger sample sizes to enhance the robustness of the conclusions drawn.

## 5 Conclusion

Our results demonstrated the prominent role of targeted therapies and immunotherapies for HER-2 breast cancer. Trastuzumab-based regimens demonstrated superior efficacy compared to other treatment options, while maintaining a comparable safety profile. Based on these findings, trastuzumab-containing regimens emerge as a preferable and recommended choice in clinical practice for managing HER2-positive breast cancer.

## Data availability statement

The original contributions presented in the study are included in the article/**Supplementary Material**. Further inquiries can be directed to the corresponding authors.

## Author contributions

SG: Conceptualization, Data curation, Writing – original draft. YL: Formal Analysis, Methodology, Software, Writing – original draft. YH: Formal Analysis, Investigation, Methodology, Writing – original draft. WL: Writing – review & editing, Conceptualization, Data curation, Validation, Visualization. KL: Visualization, Writing – review & editing, Project administration, Software.
