# Supplementary material for: Comparative efficacy and safety of targeted therapy and immunotherapy for HER2-positive breast cancer: a systematic review and network meta-analyses
Source: Front Oncol. 2024 Apr 3;14:1331055. doi: 10.3389/fonc.2024.1331055 (PMC11021689; doi:10.3389/fonc.2024.1331055)
Supplement: Supplementary file 1 [file DataSheet_1.pdf]

Supplementary Information

Comparative efficacy and safety of targeted therapy and immunotherapy for HER2-positive breast cancer: a systematic review and network meta-analyses

Suyu Gu[1,2#], Yuting Liu[3#], Yufan Huang[1#], Wenzheng Lin[1,2*], Ke Li[1,2*]

[1] Institute of Translational Medicine, Medical College, Yangzhou University, Yangzhou, China.

[2] Affiliated Hospital of Yangzhou University, Yangzhou University, Yangzhou, China.

[3] Shenyang Traditional Chinese Medicine Hospital, Shenyang, China.

[#] These authors contributed equally to this study.

[*] Corresponding authors: W.L. (m13415146506@163.com); K.L. (like20180401@163.com)

Table S1. Baseline characteristics of patients in the trials included in the meta-analysis

Author	Treatment regimen	No. of patients	Age (median, range, y)	ECOG PS (0/1/2)	Median follow-up (m)
Goss PE	Lapatinib	1571	51(24-87)	NA	12
	Placebo	1576	52(22-82)	NA	12
Cortés J	Afatinib	78	53(43-58)	16/50/12	3
	Investigator's choice of treatment	43	51(44-63)	8/27/8	3
Rimawi MF	Afatinib	10	50.3(25-88)	NA	48
	Lapatinib/Trastuzumab	19	50.3(25-88)	NA	48
Bonnefoi H	Docetaxel+trastuzumab+lapatinib	76	49.9(27.3-68.5)	NA	36
	Docetaxel+lapatinib/trastuzumab	52	49.4(27.3-70.8)	NA	36
Gianni L	Trastuzumab/Pertuzumab+Docetaxel	191	50.9±9.84	NA	24
	Trastuzumab+Pertuzumab	94	49.7±10.67	NA	24
Chan A	Neratinib	1420	52(45-59)	NA	24
	Placebo	1420	52(45-60)	NA	24
Carey LA	Paclitaxel+Trastuzumab+Lapatinib	118	50(30-75)	107/9/2	4
	Paclitaxel + Trastuzumab	64	50(25-74)	60/3/1	4
Piccart-Gebhart M	Lapatinib/trastuzumab	4197	51(19-82)	NA	12
	Trastuzumab+Lapatinib	2093	51(22-80)	NA	12
Harbeck H	Afatinib+Vinorelbine	337	51.8±11.3	202/128/1	34
	Trastuzumab+vinorelbine	169	53.1±12.3	101/66/1	34
Dieci M V	Neratinib+ paclitaxel	326	54.0(24-75)	NA	46
	Trastuzumab+paclitaxel	324	59.5(28-75)	NA	46
Awada A	Neratinib+paclitaxel	242	54.1(11.3	NA	63

			)		
	Trastuzumab+paclitaxel	237	54.1(11.3)	NA	63
Martin M	Neratinib	1420	52(45-59)	NA	6
	Placebo	1420	52(45-60)	NA	6
Dieras V	T-DM1	495	53(24-83)	312/176/8	24
	Lapatinib+Capecitabine	496	53(25-84)	299/194/2	24
Krop IE	T-DM1	404	54(28-85)	82/101/15	24
	Treatment of physician's choice	198	53(27-89)	180/200/22	24
Hurvitz SA	Trastuzumab + Pertuzumab	222	50(42-57)	209/14/0	6
	T-DM1+ Pertuzumab	221	49(41-57)	212/9/0	6
Takano T	Trastuzumab+ capecitabine	43	57(34-81)	23/18/2	24
	Lapatinib+capecitabine	43	59(37-78)	31/12/0	24
Hurvitz SA	T-DM1 + Pertuzumab	221	49.9±10.9	NA	18
	Trastuzumab + Pertuzumab	214	49.9±10.9	NA	18
Iwata H	Neratinib	1420	52(26-81)	NA	12
	Placebo	1420	53(27-72)	NA	12
Ma F	Pyrotinib+capecitabine	65	48(40-55)	NA	3
	Lapatinib+capecitabine	63	48(40-55)	NA	3
Perez EA	T-DM1+Pertuzumab/ Pertuzumab-Placebo	365	53.8	NA	4
	Trastuzumab+Taxane	367	53.8	NA	4
Tolaney SM	Abemaciclib+ Trastuzumab	158	55(47-62)	NA	36
	Trastuzumab+ Chemotherapy	79	54(47-62)	NA	36
Emens LA	T-DM1+ Atezolizumab	133	54(48-60)	93/39/1	29
	T-DM1 + Placebo	69	55(48-62)	40/28/1	29
Seligmann JF	Lapatinib+capecitabine	16	51.7±9.46	6/9/1	5.6
	Trastuzumab+capecitabine	14	51.7±9.46	13/14/3	5.6
Rugo HS	Margetuximab+	266	55(29-83)	149/11	5

	chemotherapy			7/0	
	Trastuzumab+ Chemotherapy	270	56(27-86)	161/109/0	5
Huang CS	T-DM1	743	49 (23–80)	613/130	64
	Trastuzumab	743	49 (24–79)	597/146	64
Cortés J	Trastuzumab deruxtecan	261	54.3(27.9-83.1)	154/106	33
	T-DM1	263	54.2(20.2-83.0)	175/87	33
Hua X	Endocrine therapy + trastuzumab	196	50 (45–57)	NA	36
	Chemotherapy+ trastuzumab	196	49 (42–55)	NA	36
Nuciforo P	Lapatinib alone/trastuzumab alone	154	50.0(28-79)	NA	5
	Trastuzumab + Lapatinib	149	49.0(23-77)	NA	5
Author	Treatment regimen	No. of patients	Age (median, range, y)	ECOG PS (0/1/2)	Median follow-up (m)
Goss PE	Lapatinib	1571	51(24-87)	NA	12
	Placebo	1576	52(22-82)	NA	12
Cortés J	Afatinib	78	53(43-58)	16/50/12	3
	Investigator's choice of treatment	43	51(44-63)	8/27/8	3
Rimawi MF	Afatinib	10	50.3(25-88)	NA	48
	Lapatinib/Trastuzumab	19	50.3(25-88)	NA	48
Bonnefoi H	Docetaxel+trastuzumab+lapatinib	76	49.9(27.3-68.5)	NA	36
	Docetaxel+lapatinib/trastuzumab	52	49.4(27.3-70.8)	NA	36
Gianni L	Trastuzumab/Pertuzumab+Docetaxel	191	50.9±9.84	NA	24
	Trastuzumab+Pertuzumab	94	49.7±10.67	NA	24
Chan A	Neratinib	1420	52(45-59)	NA	24
	Placebo	1420	52(45-60)	NA	24
Carey LA	Paclitaxel+Trastuzuma	118	50(30-75)	107/9/	4

	b+Lapatinib			2	
	Paclitaxel + Trastuzumab	64	50(25-74)	60/3/1	4
Piccart-Gebhart M	Lapatinib/trastuzumab	4197	51(19-82)	NA	12
	Trastuzumab+Lapatinib	2093	51(22-80)	NA	12
Harbeck H	Afatinib+Vinorelbine	337	51.8±11.3	202/128/1	34
	Trastuzumab+vinorelbine	169	53.1±12.3	101/66/1	34
Dieci M V	Neratinib+ paclitaxel	326	54.0(24-75)	NA	46
	Trastuzumab+paclitaxel	324	59.5(28-75)	NA	46
Awada A	Neratinib+paclitaxel	242	54.1(11.3)	NA	63
	Trastuzumab+paclitaxel	237	54.1(11.3)	NA	63
Martin M	Neratinib	1420	52(45-59)	NA	6
	Placebo	1420	52(45-60)	NA	6
Dieras V	T-DM1	495	53(24-83)	312/176/8	24
	Lapatinib+Capecitabine	496	53(25-84)	299/194/2	24
Krop IE	T-DM1	404	54(28-85)	82/101/15	24
	Treatment of physician's choice	198	53(27-89)	180/200/22	24
Hurvitz SA	Trastuzumab + Pertuzumab	222	50(42-57)	209/14/0	6
	T-DM1+ Pertuzumab	221	49(41-57)	212/9/0	6
Takano T	Trastuzumab+ capecitabine	43	57(34-81)	23/18/2	24
	Lapatinib+capecitabine	43	59(37-78)	31/12/0	24
Hurvitz SA	T-DM1 + Pertuzumab	221	49.9±10.9	NA	18
	Trastuzumab + Pertuzumab	214	49.9±10.9	NA	18
Iwata H	Neratinib	1420	52(26-81)	NA	12
	Placebo	1420	53(27-72)	NA	12
Ma F	Pyrotinib+capecitabine	65	48(40-55)	NA	3
	Lapatinib+capecitabine	63	48(40-55)	NA	3

Perez EA	T-DM1+Pertuzumab/ Pertuzumab-Placebo	365	53.8	NA	4
	Trastuzumab+Taxane	367	53.8	NA	4
Tolaney SM	Abemaciclib+ Trastuzumab	158	55(47-62)	NA	36
	Trastuzumab+ Chemotherapy	79	54(47-62)	NA	36
Emens LA	T-DM1+ Atezolizumab	133	54(48-60)	93/39/ 1	29
	T-DM1 + Placebo	69	55(48-62)	40/28/ 1	29
Seligmann JF	Lapatinib+capecitabine	16	51.7±9.46	6/9/1	5.6
	Trastuzumab+capecita bine	14	51.7±9.46	13/14/ 3	5.6
Rugo HS	Margetuximab+ chemotherapy	266	55(29-83)	149/11 7/0	5
	Trastuzumab+ Chemotherapy	270	56(27-86)	161/10 9/0	5
Huang CS	T-DM1	743	49 (23–80)	613/13 0	64
	Trastuzumab	743	49 (24–79)	597/14 6	64
Cortés J	Trastuzumab deruxtecan	261	54.3(27.9 -83.1)	154/10 6	33
	T-DM1	263	54.2(20.2 -83.0)	175/87	33
Hua X	Endocrine therapy + trastuzumab	196	50 (45–57)	NA	36
	Chemotherapy+ trastuzumab	196	49 (42–55)	NA	36
Nuciforo P	Lapatinib alone/trastuzumab alone	154	50.0(28-7 9)	NA	5
	Trastuzumab + Lapatinib	149	49.0(23-7 7)	NA	5

Abbreviation: No., number; ECOG PS, Eastern Cooperative Oncology Group Performance Status; y, year; T-DM1, trastuzumab emtansine; NA, not available; m, month.

Table S2. Leaguetable of overall survival(OS).

Treatments (Overall survival, HR, 95%Crl)	Afatinib	Afatinib+CT	Atezolizumab+tdm1	Capecitabine+Trastuzumab	Lapatinib	Lapatinib+Capecitabine	Lapatinib+CT	Lapatinib+Trastuzumab	Margetuximab+CT	Neratinib	Pertuzumab+Trastuzumab	placebo	Pyrotinib+Capecitabine	Trastuzumab	Trastuzumab+CT	Trastuzumab+ET	tdm1	tdm1+Pertuzumab	T-DXd	tpc
Afatinib		0.62(0.32,1.18)	0.45(0.2,1.02)	0.26(0.1,0.73)	0.58(0.27,1.23)	0.46(0.24,0.85)	0.73(0.32,1.64)	0.34(0.16,0.73)	0.85(0.43,1.68)	0.56(0.24,1.27)	0.65(0.22,1.91)	0.58(0.26,1.31)	0.31(0.14,0.72)	0.42(0.21,0.88)	0.76(0.4,1.43)	0.92(0.47,1.81)	0.61(0.33,1.11)	0.65(0.33,1.29)	1.1(0.53,2.31)	0.79(0.45,1.38)
Afatinib+CT			0.73(0.38,1.39)	0.43(0.18,1.03)	0.94(0.53,1	0.74(0.52,1.05)	1.18(0.67,2	0.55(0.31,0.98)	1.38(0.97,1.97)	0.9(0.47,1.	1.06(0.43,2.63)	0.95(0.51,1	0.51(0.27,0.97)	0.69(0.41,1	1.23(0.95,1	1.5(1.06,2.	0.98(0.72,1	1.06(0.74,1.51)	1.79(1.04,3	1.28(0.91,1

				.64)		.08)			73)		.77)		.15)	.58)	12)	.35)		.06)	.79)
**Atezolizumab+tdm1**			0.59 (0.22, 1.59)	1.28 (0.62, 2.67)	1.01 (0.56, 1.82)	1.62 (0.73, 3.58)	0.76 (0.36, 1.58)	1.89 (0.99, 3.65)	1.24 (0.56, 2.75)	1.45 (0.5, 4.21)	1.3 (0.59, 2.85)	0.7 (0.31, 1.56)	0.94 (0.47, 1.89)	1.69 (0.92, 3.1)	2.05 (1.07, 3.94)	1.35 (0.77, 2.37)	1.45 (0.75, 2.8)	2.45 (1.21, 5.01)	1.75 (0.96, 3.2)
**Capecitabine+Trastuzumab**				2.19 (0.85, 5.61)	1.72 (0.77, 3.85)	2.76 (1.02, 7.4)	1.28 (0.5, 3.33)	3.21 (1.33, 7.84)	2.1 (0.78, 5.72)	2.47 (0.73, 8.4)	2.21 (0.83, 5.92)	1.19 (0.45, 3.15)	1.61 (0.64, 4.02)	2.86 (1.23, 6.73)	3.49 (1.45, 8.46)	2.29 (1.01, 5.2)	2.46 (1.02, 6.01)	4.17 (1.65, 10.59)	2.98 (1.28, 6.95)
**Lapatinib**					0.79 (0.48, 1.29)	1.26 (0.61	0.59 (0.42, 0.83)	1.47 (0.83, 2.69	0.96 (0.69	1.13 (0.41, 3.11)	1.01 (0.76	0.54 (0.26, 1.13)	0.74 (0.59	1.31 (0.78	1.6 (0.91,	1.05 (0.66	1.13 (0.63, 2.0	1.91 (1.01	1.36 (0.82

			,2.6)		2)	,1.34)		,1.34)		,0.92)	,2.2)	2.83)	,1.67)	1)	,3.6)	,2.27)
**Lapatinib+Capecitabine**			1.6(0.9,2.86)	0.75(0.45,1.23)	1.87(1.29,2.72)	1.22(0.68,2.21)	1.43(0.57,3.58)	1.28(0.73,2.26)	0.69(0.4,1.19)	0.93(0.6,1.44)	1.66(1.26,2.2)	2.03(1.4,2.93)	1.33(1.14,1.56)	1.43(0.98,2.09)	2.42(1.53,3.85)	1.73(1.32,2.26)
**Lapatinib+CT**				0.47(0.22,0.97)	1.17(0.66,2.06)	0.76(0.34,1.69)	0.9(0.33,2.46)	0.8(0.37,1.75)	0.43(0.19,0.96)	0.58(0.29,1.16)	1.04(0.63,1.73)	1.27(0.73,2.22)	0.83(0.48,1.46)	0.89(0.51,1.57)	1.51(0.75,3.06)	1.08(0.6,1.94)
**Lapatinib+Trastuzumab**					2.5(1.4,4.5)	1.64(1.0	1.92(0.69,5.34)	1.72(1.1	0.92(0.44,1.9	1.25(0.9	2.23(1.3	2.72(1.5	1.79(1.1	1.92(1.07,3.	3.24(1.7	2.32(1.3

			2,2.64)		,2.69)	4)	7,1.61)	1,3.78)	2,4.85)	1,2.87)	46)	,6.16)	8,3.91)
**Margetuximab+CT**			0.65(0.34,1.27)	0.77(0.31,1.9)	0.69(0.36,1.3)	0.37(0.19,0.71)	0.5(0.29,0.84)	0.89(0.7,1.14)	1.09(0.77,1.53)	0.71(0.51,1)	0.77(0.54,1.09)	1.3(0.74,2.25)	0.93(0.63,1.36)
**Neratinib**				1.17(0.4,3.42)	1.05(0.88,1.25)	0.56(0.25,1.26)	0.76(0.51,1.14)	1.36(0.74,2.51)	1.66(0.86,3.21)	1.09(0.62,1.93)	1.17(0.6,2.28)	1.98(0.97,4.06)	1.42(0.77,2.6)
**Pertuzumab+Trastuzumab**					0.9(0.3	0.48(0.17,1.	0.65(0.	1.16(0.	1.42(0.	0.93(0.	1(0.43,2.3	1.69(0.	1.21(0.

**ab**		1,2.57)	4)	24,1.75)	48,2.78)	57,3.49)	38,2.3)	1)	62,4.63)	48,3.03)
**placebo**			0.54(0.25,1.18)	0.73(0.5,1.05)	1.3(0.72,2.34)	1.58(0.84,2.99)	1.04(0.6,1.79)	1.12(0.58,2.12)	1.89(0.94,3.78)	1.35(0.75,2.42)
**Pyrotinib+Capecitabine**				1.35(0.67,2.71)	2.41(1.3,4.45)	2.94(1.53,5.68)	1.93(1.09,3.41)	2.07(1.07,4.02)	3.51(1.72,7.18)	2.51(1.36,4.61)
**Trastuzumab**					1.78(1	2.17(1	1.43(0	1.53(0.9,	2.6(1.	1.85(1

		.12,2.84)	.29,3.66)	.96,2.14)	2.6)	43,4.68)	.18,2.93)
**Trastuzumab+CT**			1.22(0.96,1.54)	0.8(0.64,1.01)	0.86(0.67,1.11)	1.45(0.89,2.38)	1.04(0.78,1.4)
**Trastuzumab+ET**				0.66(0.47,0.91)	0.71(0.5,0.99)	1.19(0.69,2.06)	0.85(0.58,1.25)
**tdm1**					1.07(0.	1.82(	1.3(1

		76, 1.51)	1.18, 2.81)	.05, 1.61)
tdm1+Pertuzumab			1.69 (0.97, 2.95)	1.21 (0.82, 1.78)
T-DXd				0.72 (0.44, 1.16)
tpc				

Table S3. Leaguetable of pathologic complete response (pCR).

Treatments(pCR, OR, 95%Crl)	Lapatinib+chemical therapy	Pertuzumab+chemical therapy	Pertuzumab+Trastuzumab	Trastuzumab+chemical therapy	Trastuzumab emtansine+Pertuzumab
**Lapatinib+chemical therapy**		1.18 (0.56, 2.46)	0.75 (0.35, 1.6)	1.53 (1.06, 2.24)	0.75 (0.33, 1.67)
**Pertuzumab+chemical therapy**			0.64 (0.32, 1.28)	1.3 (0.69, 2.46)	0.64 (0.3, 1.34)
**Pertuzumab+Trastuzumab**				2.03 (1.06, 3.99)	1 (0.77, 1.3)
**Trastuzumab+chemical therapy**					0.49 (0.24, 0.99)
**Trastuzumab emtansine+Pertuzumab**					

Table S4. Leaguetable of adverse events (AEs).

Treatments	abe_t	afa	afa_CT	ate_tdm1	c_t	l	l_c	l_CT	l_t	m_CT	n	ner_CT	p_CT	p_t	placebo	py_c	t	t_CT	t_ET	tdm1	tdm1_p	TDXd	tpc
**abe_t**		0.59(0.18,1	1.19(0.46,3	1.57(0.5,4.	0.23(0.06,0	0.93(0.34,2	0.83(0.31,2	1.16(0.44,2	1.13(0.41,3	0.67(0.25,1	1.23(0.42,3	1.7(0.67,4.	0.95(0.32,2	0.78(0.3,1.	0.71(0.25,1	1.56(0.46,5	0.44(0.16,1	0.74(0.31,1	0.11(0.04,0	0.75(0.29,1	0.81(0.32,1	0.81(0.29,2	0.66(0.24,1

	.9)	.02)	88)	.87)	.46)	.17)	.97)	.01)	.75)	.5)	18)	.73)	96)	.97)	.24)	.16)	.73)	.33)	.87)	.99)	.21)	.74)
afa		2.01(0.91,4.54)	2.67(0.95,7.64)	0.38(0.1,1.37)	1.57(0.66,3.79)	1.41(0.6,3.37)	1.98(0.8,4.92)	1.92(0.81,4.63)	1.15(0.45,2.91)	2.09(0.82,5.42)	2.9(1.23,6.86)	1.62(0.58,4.44)	1.32(0.55,3.19)	1.2(0.48,3.05)	2.65(0.88,8.26)	0.74(0.32,1.77)	1.26(0.56,2.84)	0.19(0.07,0.55)	1.28(0.58,2.87)	1.37(0.59,3.19)	1.37(0.55,3.47)	1.12(0.52,2.47)
afa_CT			1.32(0.59,3.02)	0.19(0.06,0.57)	0.78(0.43,1.4)	0.7(0.41,1.23)	0.98(0.56,1.71)	0.95(0.53,1.71)	0.57(0.32,1.01)	1.04(0.52,2.07)	1.43(0.89,2.31)	0.8(0.38,1.64)	0.65(0.38,1.11)	0.6(0.31,1.15)	1.32(0.53,3.31)	0.37(0.21,0.65)	0.62(0.43,0.91)	0.1(0.04,0.2)	0.63(0.4,1.01)	0.68(0.42,1.09)	0.68(0.36,1.3)	0.56(0.32,0.95)
ate_tdm1				0.14(0.04,	0.59(0.27,	0.53(0.25,	0.74(0.31,	0.72(0.33,	0.43(0.18,	0.79(0.33,	1.09(0.48,	0.61(0.23,	0.5(0.22,1	0.45(0.22,1	1(0.36,2.7	0.28(0.13,	0.47(0.22,	0.07(0.03,	0.48(0.24,	0.51(0.24,	0.52(0.23,	0.42(0.19,

	0.47)	1.25)	1.09)	1.71)	1.53)	1.01)	1.81)	2.37)	1.55)	.08)	.02)	8)	0.58)	0.98)	0.19)	0.92)	1.07)	1.13)	0.9)
**c_t**		4.09(1.43,12.5)	3.65(1.45,10.06)	5.14(1.69,16.77)	5.01(1.75,15.28)	2.98(0.97,9.81)	5.44(1.8,17.61)	7.52(2.57,23.53)	4.2(1.26,14.74)	3.43(1.18,10.7)	3.13(1.05,9.97)	6.91(2.14,23.92)	1.93(0.69,5.87)	3.26(1.16,9.89)	0.5(0.15,1.82)	3.31(1.25,9.59)	3.56(1.26,10.8)	3.57(1.2,11.24)	2.93(1.02,9.01)
**l**			0.89(0.55,1.46)	1.26(0.67,2.36)	1.22(1.08,1.38)	0.73(0.38,1.4)	1.33(0.92,1.92)	1.84(1.05,3.23)	1.03(0.47,2.2)	0.84(0.48,1.46)	0.77(0.56,1.04)	0.69(0.72,4.04)	0.47(0.41,0.54)	0.8(0.49,1.29)	0.12(0.05,0.28)	0.81(0.56,1.18)	0.87(0.53,1.42)	0.87(0.49,1.56)	0.71(0.42,1.21)
**l_c**				1.4(0.76	1.37(0.8	0.81(0.4	1.49(0.8	2.05(1.2	1.15(0.5	0.94(0.5	0.86(0.4	1.89(0.9	0.53(0.3	0.89(0.5	0.14(0.0	0.91(0.6	0.97(0.6	0.97(0.5	0.8(0.49

		,2.59)	4,2.22)	3,1.53)	1,2.73)	,3.51)	4,2.42)	5,1.59)	8,1.52)	3,3.87)	3,0.84)	7,1.4)	6,0.3)	6,1.25)	2,1.54)	6,1.69)	,1.31)
**l_CT**			0.97(0.52,1.83)	0.58(0.32,1.05)	1.06(0.51,2.2)	1.46(0.89,2.42)	0.82(0.38,1.71)	0.67(0.38,1.17)	0.61(0.3,1.23)	1.34(0.53,3.46)	0.38(0.2,0.7)	0.64(0.42,0.96)	0.1(0.04,0.21)	0.65(0.39,1.09)	0.69(0.42,1.15)	0.69(0.35,1.37)	0.57(0.31,1.06)
**l_t**				0.6(0.31,1.15)	1.09(0.74,1.6)	1.5(0.86,2.64)	0.84(0.38,1.8)	0.68(0.39,1.19)	0.63(0.45,0.87)	1.38(0.59,3.3)	0.39(0.34,0.44)	0.65(0.4,1.06)	0.1(0.04,0.22)	0.66(0.46,0.96)	0.71(0.43,1.16)	0.71(0.4,1.28)	0.58(0.35,0.99)
**m_CT**					1.83(0.	2.52(1.	1.41(0.	1.15(0.	1.05(0.	2.32(0.	0.65(0.	1.1(0.7	1.17(0.	1.12(0.	1.19(0.	1.2(0.5	0.98(0.

		87,3.88)	49,4.31)	65,3.03)	64,2.07)	51,2.17)	89,6.07)	34,1.23)	1,1.71)	07,0.37)	65,1.93)	7,2.05)	9,2.42)	52,1.87)
n			1.38(0.71,2.71)	0.77(0.33,1.8)	0.63(0.32,1.23)	0.58(0.47,0.7)	1.27(0.5,3.26)	0.36(0.24,0.53)	0.6(0.33,1.1)	0.09(0.04,0.22)	0.61(0.36,1.03)	0.65(0.35,1.21)	0.65(0.33,1.3)	0.54(0.28,1.02)
**ner_CT**				0.56(0.28,1.1)	0.46(0.28,0.73)	0.42(0.22,0.79)	0.92(0.38,2.26)	0.26(0.15,0.44)	0.43(0.32,0.58)	0.07(0.03,0.13)	0.44(0.29,0.68)	0.47(0.31,0.72)	0.47(0.26,0.88)	0.39(0.22,0.67)
**p_CT**					0.81(0	0.74(0	1.64(0	0.46(0	0.78(0	0.12(0	0.79(0	0.85(0	0.85(0	0.7(0.

		.44,1.54)	.33,1.72)	.58,4.67)	.22,1)	.42,1.48)	.05,0.3)	.4,1.58)	.45,1.62)	.38,1.93)	33,1.5)
**p_t**			0.91(0.48,1.73)	2.01(0.83,4.93)	0.56(0.33,0.97)	0.95(0.65,1.4)	0.15(0.07,0.31)	0.97(0.64,1.48)	1.04(0.78,1.39)	1.04(0.56,1.92)	0.85(0.49,1.48)
**placebo**				2.21(0.89,5.56)	0.62(0.44,0.87)	1.04(0.59,1.85)	0.16(0.07,0.38)	1.06(0.66,1.72)	1.14(0.64,2.03)	1.14(0.59,2.2)	0.94(0.51,1.72)
**py_c**					0.28(	0.47(	0.07(	0.48(	0.52(	0.52(	0.42(

		0.12,0.65)	0.2,1.1)	0.02,0.21)	0.22,1.05)	0.22,1.2)	0.21,1.26)	0.18,1.01)
**t**			1.69(1.06,2.68)	0.26(0.11,0.58)	1.71(1.22,2.43)	1.84(1.14,2.95)	1.84(1.05,3.25)	1.51(0.91,2.52)
**t_CT**				0.16(0.08,0.29)	1.02(0.74,1.4)	1.09(0.8,1.48)	1.09(0.63,1.89)	0.9(0.56,1.42)
**t_ET**					6.57	7.03	7.06	5.8(

		(3.22,14.24)	(3.48,15.17)	(3.04,17.21)	2.63,13.42)
**tdm1**			1.07(0.77,1.49)	1.07(0.69,1.68)	0.88(0.6,1.28)
**tdm1_p**				1(0.58,1.74)	0.82(0.51,1.33)
**TD**					0.

**Xd**		82(0.46,1.47)
**tpc**		
